# The Study of Amorphous Kaempferol Dispersions Involving FT-IR Spectroscopy

**DOI:** 10.3390/ijms242417155

**Published:** 2023-12-05

**Authors:** Natalia Rosiak, Ewa Tykarska, Judyta Cielecka-Piontek

**Affiliations:** 1 Department of Pharmacognosy and Biomaterials, Faculty of Pharmacy, Poznan University of Medical Sciences, 3 Rokietnicka St., 60-806 Poznan, Poland; nrosiak@ump.edu.pl; 2 Department of Chemical Technology of Drugs, Poznan University of Medical Sciences, 6 Grunwaldzka St., 60-780 Poznan, Poland; etykarsk@ump.edu.pl

**Keywords:** amorphous solid dispersion, kaempferol, FT-IR, PCA

## Abstract

Attenuated total reflection-Mid-Fourier transform-infrared (ATR-Mid-FT-IR) spectroscopy combined with principal component analysis (PCA) has been applied for the discrimination of amorphous solid dispersion (ASD) of kaempferol with different types of Eudragit (L100, L100-55, EPO). The ASD samples were prepared by ball milling. Training and test sets for PCA consisted of a pure compound, physical mixture, and incomplete/complete amorphous solid dispersion. The obtained results confirmed that the range 400–1700 cm^−1^ was the major contributor to the variance described by PC1 and PC2, which are the fingerprint region. The obtained PCA model selected fully amorphous samples as follows: five for KMP-EL100, two for KMP-EL100-55, and six for KMP-EPO (which was confirmed by the XRPD analysis). DSC analysis confirmed full miscibility of all ASDs (one glass transition temperature). FT-IR analysis confirmed the formation of hydrogen bonds between the –OH and/or –CH groups of KMP and the C=O group of Eudragits. Amorphization improved the solubility of kaempferol in pH 6.8, pH 5.5, and HCl 0.1 N.

## 1. Introduction

Kaempferol (KMP) is a flavonoid found in various edible plants (red fruits, grapes, citrus fruits), and it has also been identified in several medicinal plants (*Hippophae rhamnoides*, *Tilia* spp., *Ginkgo biloba*). As confirmed by numerous preclinical and clinical studies, KMP has been noted for its ability to exhibit antioxidant, anti-inflammatory, anticancer, and neuroprotective properties [1,2,3,4,5]. Nevertheless, KMP struggles to reach the necessary therapeutic concentrations. This is related to the limited water solubility of KMP, which results in poor bioavailability after oral administration [6,7]. Studies have shown that upon absorption KMP is frequently metabolized into sulfate, methyl, or glucuronide forms [7,8]. To boost absorption and improve the clinical response, increasing the solubility of KMP is essential.

Obtaining amorphous solid dispersion (ASD) is a potent method for enhancing the solubility and bioavailability of poorly water-soluble compounds [9,10,11,12,13]. ASD involves the dispersion of the drug (polyphenol) within an amorphous matrix, typically a hydrophilic polymer or a combination of polymers. Transforming the crystalline polyphenol into an amorphous form significantly enhances the dissolution rate and solubility, resulting in improved absorption and therapeutic effects. The amorphous matrix provides stability and prevents the polyphenol from reverting to its crystalline form, ensuring sustained supersaturation and improved drug release [9,10,11,12,14,15]. Obtaining ASD can be achieved through various techniques such as hot melt extrusion [11], spray drying [16,17], solvent evaporation [18], and ball milling [9,10,19]. These methods allow for achieving a molecular-level dispersion of polyphenols within the matrix, creating a highly homogeneous and amorphous system. In recent years, it has been demonstrated that compounds such as sinapic acid [18], resveratrol [17], pterostilbene [10], curcumin [17,20,21], hesperidin [9,22], daidzein [16], genistein [19,23], hesperetin [12,22], naringenin [24], and quercetin [25,26,27] can be can be formulated using ASD. To date, no ASDs of KMP have been reported.

For ASD analysis, X-ray powder diffraction (XRPD) is the method of choice, confirming the absence of crystallinity through the amorphous halo in the X-ray powder diffraction pattern [9,10,11,12,28]. Another useful technique is differential scanning calorimetry (DSC), where the disappearance of the compound’s melting point indicates its amorphous state. Nevertheless, efforts are being made to identify a rapid analytical method capable of distinguishing both crystalline and amorphous forms of active pharmaceutical ingredients (APIs) simultaneously.

Recently, vibrational spectroscopic techniques have shown promise as fast, nondestructive, and simple methods [29,30]. According to the literature, FT-IR spectroscopy combined with multivariate analysis (especially principal component analysis, PCA) was confirmed to be effective in classifying, among others, the style and quality of sparkling wines [31], saffron samples in terms of geographical origin [32], authentication and adulteration detection of Iranian saffron samples [33], differences in ewe’s milk [34], detection of the adulteration of cocoa content in chocolates [35], and identify different parts and harvests time of *Dendrobium officinale* [36]. 

Culbert et al. [31] demonstrated the effective classification of sparkling wine style and quality by employing ATR-MIR spectroscopy in conjunction with PCA. The models developed in this study exhibited high accuracy and good predictive ability for both style and quality ratings. Maamouri et al. [34] studied the effect of the lactation period and feed type on the quality of ewe’s milk. In their study, applying PCA to the spectral ranges of 2800–3000 cm^−1^ and 900–1500 cm^−1^ enabled effective visual discrimination of milk samples based on the feed.

Therefore, our research aimed to employ infrared analysis combined with chemometric methods (i.e., PCA) to develop a model for detecting the amorphous state of KMP within the Eudragit matrix.

## 2. Results and Discussion

This paper shows the screening of ASD of KMP with different types of Eudragit (EL100, EL100-55, EPO). To the best of our knowledge, this work is the first report using PCA combined with FT-IR spectroscopy as an efficient method to confirm the amorphous state of KMP in a polymer matrix.

The first stage of the study focused on preparing training and test sets for PCA analysis. Incomplete (NA) and complete (A or ASD) amorphous solid dispersions (confirmed by XRPD) were obtained by the milling method. Physical mixtures (PM or ph.m.) were prepared using the vortex method. The total amount of twenty-five samples were prepared and analyzed by XRPD and FT-IR. From the registered FT-IR spectra, 18 constituted a training set, while 7 constituted a test set (see Table 1).

**Figure 1:**
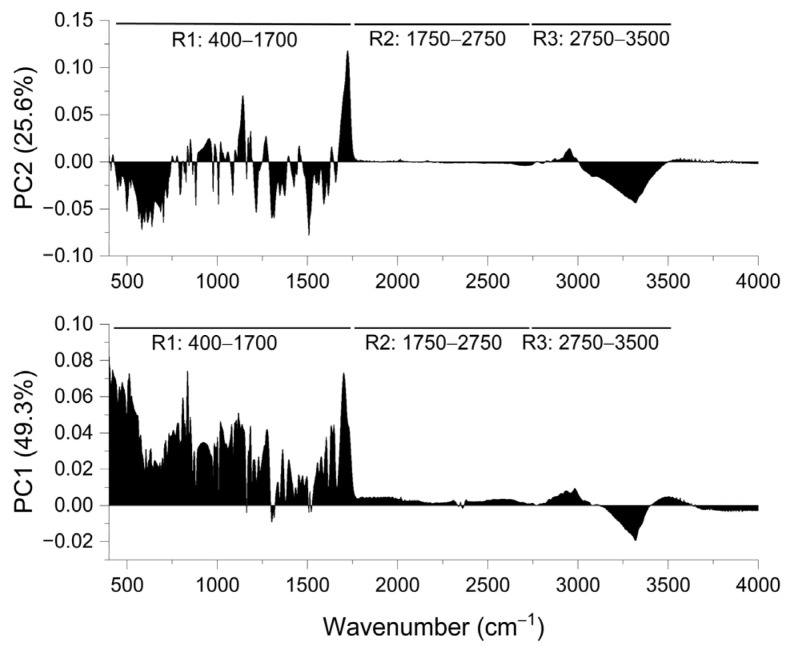
Loading plot of PC1 and PC2. R1, R2, R3—range of the wavenumber.

In the next step, the obtained spectra data were analyzed using PCA. A loading plot of the first and second principal component (PC1 and PC2) for the full MIR range (400–4000 cm^−1^) is given in Figure 1 and divided into three regions (R) as follows: R1: 400–1700 cm^−1^, R2: 1750–2750 cm^−1^, and R3: 2750–3500 cm^−1^.

In the fingerprint region (R1), PC1 showed a positive correlation, whereas PC2 showed a positive and negative correlation. The R2 region did not significantly influence the differentiation of KMP–Eudragit samples since no significant peaks were detected in that region. In the R3 region, a negative correlation prevails for PC1 and PC2. The obtained results confirmed that the major contributor to the variance described by PC1 and PC2 was the R1 region (400–1700 cm^−1^), which constitutes a fingerprint region characterized by a highly complex but unique absorption pattern for each organic structure.

**Figure 2:**
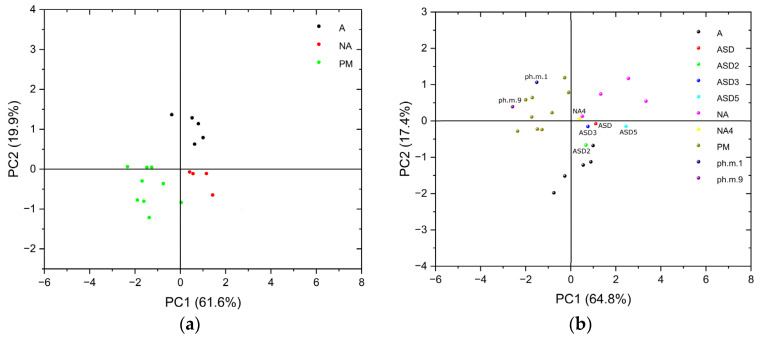
PCA score plot of the kaempferol–Eudragit samples from the wavenumber (400–1700 cm^−1^) from the (**a**) training set and (**b**) the training and test set. Samples of the “training set”: A—amorphous solid dispersion; NA—incomplete amorphous solid dispersion; PM—physical mixture. Samples of the “test set”: ASD (ASD, ASD2, ASD3, ASD5)—amorphous solid dispersion; NA (NA4)—incomplete amorphous solid dispersion; ph.m. (ph.m.1, ph.m.9)—physical mixture.

FT-IR spectra of the training set and test set (wavenumbers from the fingerprint region) were used to generate a PCA score plot. The PCA score plot of the first two principal components (PC) derived from the R1 range of all KMP–Eudragit samples is shown in Figure 2a,b.

For the training set (Figure 2a), PC1 explains 61.6% of the variation observed and resulted in a clear separation of the amorphous solid dispersions (A, black dot) from the other samples. For the training and test set (Figure 2b), PC1 explains 64.8% of the variation observed and resulted in a clear separation of the amorphous solid dispersions (A/ASD, lower right quadrant) from the other samples. Separation of physical mixtures (upper left quadrant) and incomplete amorphization (upper right quadrant) were also observed. Several outliers were observed, i.e., three physical mixtures and two amorphous solid dispersions in the lower left quadrant. Nevertheless, they are located very close to the quarters they should belong to (see Figure 2b).

**Figure 3:**
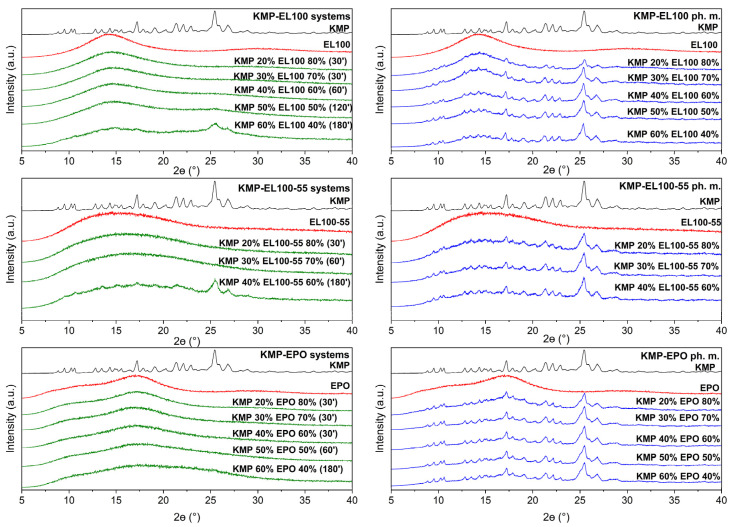
XRPD analysis: kaempferol crystalline form (black), Eudragit (red), and kaempferol–Eudragit samples after the milling process (green) and the physical mixtures (blue). Legend: KMP—kaempferol; EL100—Eudragit L100; EL100-55—Eudragit L100-55; EPO—Eudragit EPO; KMP 20–60%—content of kaempferol in the samples; 30′–180′—time of the milling process.

The obtained PCA model was employed in the subsequent part of the research to select ASDs among the samples obtained from the milling process. The diffractograms of samples selected on the basis of PCA are presented in Figure 3.

The crystalline KMP is characterized by a crystal pattern consisting of a series of well-defined sharp peaks between 8° 2Θ and 30° 2Θ. The diffractogram of L100, L100-55, and EPO (Figure 3, red line) displayed no peaks due to its amorphous nature [37,38,39]. KMP–Eudragit physical mixtures are the sum of diffraction patterns of KMP and Eudragit. 

The samples for which a complete disappearance of the Bragg peaks (“halo effect”) was observed in the diffractograms of KMP–Eudragit systems (Figure 3, green line) will be referred to as ASD. The highest efficiency of amorphization was observed in the following order: EPO, L100, and L100-55. ASDs with KMP-EPO were obtained after 30, 60, and 180 min of milling, allowing for the highest KMP loading in the polymer matrix (60% KMP in ASD). Eudragit L100 allowed the amorphous KMP to be obtained after 30, 60, and 120 min; however, it was possible to load up to 50% of the compound into the polymer matrix. The lowest amorphization efficiency was shown by EL100-55, for which ASDs were obtained for samples with a KMP content of 20% (after 30 min) and 30% (after 60 min). Regarding the systems with higher KMP loading values, incomplete amorphousness was still observed even after three hours of milling.

The obtained results confirm that Eudragits are suitable polymers for achieving the amorphous state of the drug, which is consistent in the literature. The completely amorphous “halo” of the X-ray diffraction patterns in compound-Eudragit systems were also observed by Chenchen et al. [15], Wang et al. [40], Zong et al. [14], and Alsayad et al. [41]. In addition, evidence was provided that the use of PCA together with FT-IR spectroscopy is an effective technique for confirming the amorphous state of KMPs.

Subsequently, thermal characterization of KMP, EL100, EL100-55, and EPO, and their ASDs was carried out using thermogravimetric (TG) and differential scanning calorimetry (DSC) methods. The TG analysis was carried out to determine the moisture content and sample stability upon heating. KMP was stable up to ~315 °C (moisture loss: 1.31%), EL100 to 176 °C (moisture loss: 1.45%), EL100-55 to 176 °C (moisture loss: 0.86%), and EPO to 205 °C (moisture loss: none) (Figure S1, Supplementary Materials).

TG analysis of the ASD of KMP–Eudragit confirmed that all samples degraded in the range where the melting point of kaempferol was observed (Figure S2a–c, Supplementary Materials). For this reason, in a further part of this study, the DSC analysis was carried out in terms of the stability of all ASDs and was aimed at recording the glass transition.

The DSC thermogram of KMP reveals a sharp endothermic peak at its melting point of 287 °C, indicating its crystalline nature (Figure S3, Supplementary Materials). This is consistent with reports in the literature. Colombo et al. observed the melting point of KMP at about 287 °C [42], Tzeng et al. at 285.5 °C [43], and Zhou et al. at 288.1 °C [44]. 

**Figure 4:**
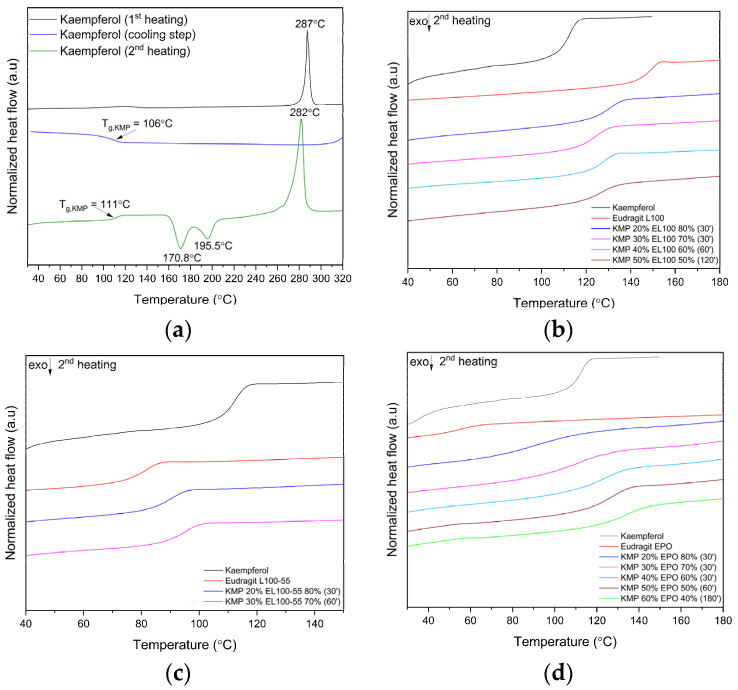
DSC analysis: (**a**) melted kaempferol; (**b**) Eudragit L100 and amorphous solid dispersion of kaempferol–Eudragit L100; (**c**) Eudragit L100-55 and amorphous solid dispersion of kaempferol–Eudragit L100-55; (**d**) Eudragit EPO and amorphous solid dispersion of kaempferol–Eudragit EPO.

The heating and cooling method allowed us to observe the glass transition of raw samples and ASDs (Figure 4a–d).

In the cooling step, a glass transition temperature (T_g_) of melted KMP was noted at 106 °C (Figure 4a, blue line). In the second heating step, three thermal effects were visible on the thermogram of the melted KMP: T_g_ at 111 °C, wide exothermic effect with maxima at 170.8 °C and 195.5 °C (probably corresponding to the cold crystallization [45]), and melting point (T_m_) at 281.8 °C (Figure 4a). The percentage of KMP crystallinity after the melting process was determined to be about 6%. The crystallinity percent was determined using the following equation (Equation (1)):

(1) $$\%\;\mathrm{Crystallinity}=\frac{\Delta H_{m}-\Delta H_{cc}}{\Delta H_{m{}^{\circ}}}\times 100\%,$$

In this equation, the heats of melting (ΔH_m_) and cold crystallization (ΔH_cc_) are in terms of J·(g·°C)^−1^. ΔH_m°_ stands as a reference value and represents the heat of melting in J·(g·°C)^−1^ if the KMP was 100% crystalline.

It is suggested that the observed glass transition is related to the presence of ~94% of the KMP amorphous phase.

Amorphous KMP produced by heat quenching in the DSC cycle exhibited a T_g_ of 111.0 °C and the amorphous polymers displayed T_g_ values of 147.1 °C, 79.7 °C and 55.4 °C for EL100, EL100-55, and EPO, respectively. Based on the obtained DSC results for raw samples, theoretical studies (see Section 3.4.2) were conducted to predict the T_g_ values of amorphous KMP–Eudragit systems.

Both the Gordon–Taylor (G-T) and Couchman–Karasz (C-K) models rely on the free volume theory. These mathematical models are applicable under the assumption that the mixing partners are perfectly blended and share similar forms and sizes. The absence of interactions between the mixing partners and the additive behavior of the partners in their free volume are indicated by these models [46]. The differences observed between the experimental (T_g,exp_) and theoretical (T_g,theo_) values are related to positive (T_g,exp_ > T_g,theo_) or negative deviations (T_g,exp_ < T_g,theo_) [10].

The experimental and predicted values for T_g_ using the G–T (T_g,G-T_) and C–K (T_g,C-K_) equations are shown in Table 2.

The fundamental determinant of amorphous pharmaceutical solid dispersion system stability is the drug–polymer miscibility; partial miscibility can lead to the creation of concentrated drug domains that may be susceptible to recrystallization after manufacture and during storage [47,48]. A single T_g_ was observed for all of the ratios of KMP–Eudragit ASDs. This suggests miscibility of KMP and polymer in the given ratios and the presence of a single phase in all of the systems. Lee et al. [49] confirmed the miscibility of ternary blends (biodegradable polymers with natural polyphenol) based on a single T_g_ value. Additionally, the literature has confirmed, based on DSC analysis, the formation of miscible systems with Eudragit EPO involving drugs such as ibuprofen, naproxen, efavirenz, and indomethacin, among others [47,50,51]. In other studies, full miscibility has been demonstrated based on the observation of a single T_g_ value for pterostilbene–Soluplus^®^ systems [10], hesperidin–Soluplus^®^/hesperidin–HPMC systems [9], curcumin–piperine–PVPVA 64 systems [11], genistein–amino acid systems [19], and hesperidin–PVP dispersions [52].

As shown in Table 2, the experimentally determined T_g_ values for all KMP-EL100 ASDs are below the T_g_ of the polymer, suggesting a plasticization effect of the KMP on the EL100. Similar results were obtained by Sathigari et al. for efavirenz–Plasdone S-630 and efavirenz-EPO binary mixtures [47]. In another study, Kanaze et al. showed that the observed T_g_ values for hesperidin–PVP dispersions were lower than that recorded for the pure polymer. They proposed that the low-molecular-mass compound acted as a plasticizer, reducing the T_g_ value of the polymer and/or some weak interaction was involved between the PVP and hesperidin, causing the formation of amorphous solid dispersion systems [52]. 

In contrast, the T_g_ values for all ASDs of KMP-EL100-55 and KMP-EPO are higher than that of pure polymer. In the literature, this is often referred to as the antiplasticization effect [53,54]. Moreover, all T_g,exp_ values for the KMP–Eudragit ASDs are less than the theoretical values. Sathigari et al. reported similar results for efavirenz–EPO systems. They suggested that the free volume in the homogenous phase is larger than that in the ideal mixture assumed by the G-T and C-K models [47].

Inquiry into the interaction and/or compatibility of polymer–polymer and polymer–drug systems have been conducted by numerous researchers using FT-IR [9,10,49,55,56]. In order to confirm the molecular interaction between KMP and Eudragits, we performed spectroscopic analyses on both pure compounds as well as KMP–Eudragit physical mixtures and KMP–Eudragit ASDs.

The experimental bands of KMP were compared with the theoretical FT-IR spectrum, which was calculated using the B3LYP/6-31G(d,p) level of theory (see Figure S4). The most characteristic bands of the crystalline KMP were observed in the FT-IR–ATR spectra at about 400–1800 cm^−1^ and 3000–3500 cm^−1^. In the first range, predominant bands correspond to the OH, CCOH, CCOC, CCO, CH, OH, and C=O vibrations. In the second range, one broad band is visible at 3321 cm^−1^, corresponding to OH groups (see Table S1, Supplementary Materials).

**Figure 5:**
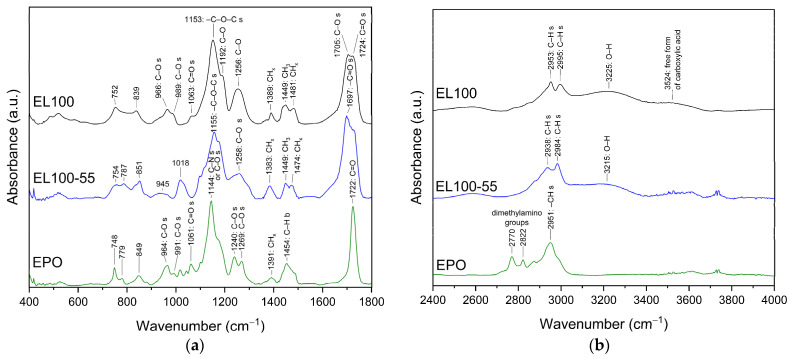
FT-IR analysis: Eudragit L100 (black line, EL100), Eudragit L100-55 (blue line, EL100-55), Eudragit EPO (green line, EPO). (**a**) Range 400–1800 cm^−1^; (**b**) range 2400–4000 cm^−1^. Legend: s—stretching; b—bending. Assignments bands were made based on values reported in the literature [35,57,58,59,60,61,62,63,64,65,66,67,68].

Assignment bands for Eudragit L100, L100-55, and EPO (Figure 5) were made based on values report in the literature (see Table S2, Supplementary Materials).

**Figure 6:**
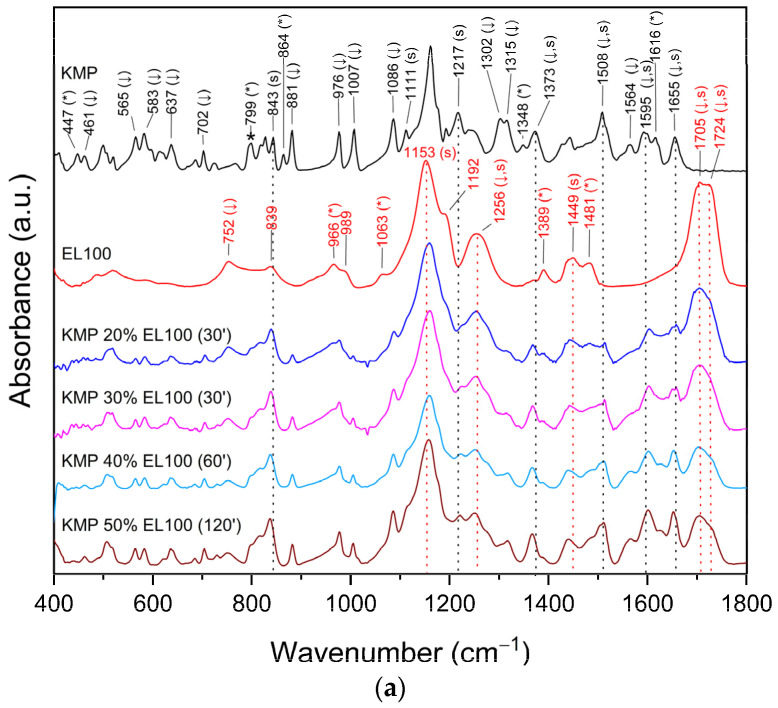

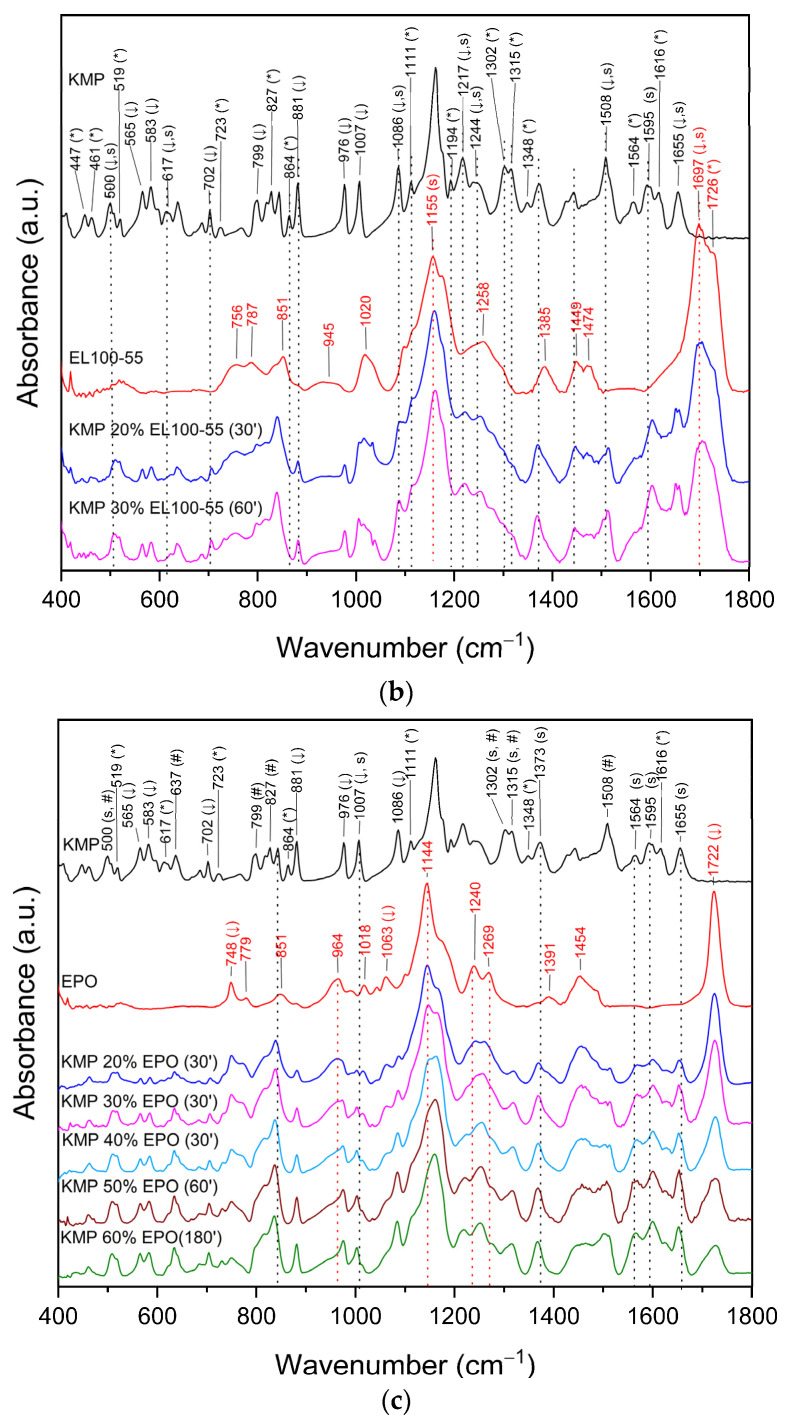
FT-IR–ATR analysis, range 400–1800 cm^−1^: (**a**) kaempferol–Eudragit L100 amorphous solid dispersion; (**b**) kaempferol–Eudragit L100-55 amorphous solid dispersion; (**c**) kaempferol–Eudragit EPO amorphous solid dispersion. Legend: kaempferol (black line, KMP); Eudragit L100/L100-55/EPO (red line, EL100/EL100-55/EPO); the percentage of KMP in the system (KMP 20%–KMP 60%); shape change (#); band disappearance (*); intensity decrease (↓); band shift (s).

Drug and polymer interactions frequently result in observable changes in FT-IR spectra [10,11,12,69]. The FT-IR spectra of KMP, EL100, EL100-55, EPO, and KMP–Eudragit ASDs, as well as physical mixtures are shown in Figure 6a–c, Figure S5a–c and Figure S6a–c. No noticeable alterations were found when the FT-IR spectra of KMP were compared to the physical mixtures, showing that all Eudragits were not involved in the intermolecular interaction with KMP in the physical mixtures (see Figure S6a–c, Supplementary Materials). In contrast, the FT-IR spectra of the KMP–Eudragit ASDs clearly showed intermolecular interactions between KMP and Eudragits (Figure 6a–c and Figure S5a–c).

In the ranges of 400–1800 cm^−1^ (Figure 6a–c) and 2600–4000 cm^−1^ (Figure S5a–c), numerous changes in the characters of bands from both KMP and Eudragits were observed (shape change—#; band disappearance—*; intensity decrease—↓; band shift—s). The observed changes are summarized in Tables S3–S5 for KMP-EL100, KMP-EL100-55, and KMP-EPO, respectively, along with the assignment of the corresponding bands. The shifting of the characteristic peaks of KMP and Eudragits in the FT-IR suggests that the chemical environment of the molecules has changed. In addition, these shifts indicate the formation of new bonds or interactions between the KMP and the polymer. A decrease in the intensity of certain FT-IR bands could indicate a reduction in the concentration of specific functional groups, whereas disappearance is a strong indicator of a chemical change or interaction. These peaks could represent specific functional groups that have interacted with the Eudragits.

In all ASDs, the most important changes involve bands corresponding to –CH (KMP-EL100: 799, 843, 1217 cm^−1^; KMP-EL100-55: 799, 827, 1111, 1217 cm^−1^), –CO (KMP-EL100/-EL100-55/-EPO: 1302, 1315, ~1600–1655 cm^−1^), and –OH (KMP-EL100: 461, 864, 3321 cm^−1^; KMP-EL100-55: ~460–519, 864, 881, 1194, 3321 cm^−1^; KMP-EPO: ~500–519, 864, 881, 3321 cm^−1^) bonds in KMP, indicating that they may be involved in the formation of hydrogen bonds with Eudragits.

In the case of KMP-EL100, a disappearance of the characteristic EL100 bands were recorded at 966 cm^−1^ (C–O or O–H) [57,58], 1063 cm^−1^ (C=O) [59], 1192 cm^−1^ (C–O) [70], and 1389 cm^−1^ (CH_x_) [63]. A decrease in intensity was observed for bands at 1481 cm^−1^ (CH_x_) [63], 1705 cm^−1^ (C–O) [67], and 1724 cm^−1^ (C=O) [12,58,65], among others. In addition, band shifts were applied to peaks at 1705 cm^−1^ and 1724 cm^−1^. On the other hand, for KMP–EL100–55, there were shifts in the bands recorded at 1155 cm^−1^ (–C–O–C) [62], 1697 cm^−1^ (COO or –C=O) [62,65], and 3215 cm^−1^ (O–H) [61]; they was a decrease in band intensity at 1697 cm^−1^ and an increase in band intensity at 3215 cm^−1^. For KMP-EPO, a decrease in intensity or a disappearance of the characteristic EPO bands were observed at 1144 cm^−1^ (C–N or C–O) [60], 1240 cm^−1^ (C–O) [71], 1269 cm^−1^ (C–O) [71], 1722 cm^−1^ (C=O) [68], 2770 cm^−1^ (dimethylamino group) [66], and 2822 cm^−1^ (dimethylamino group) [66].

The obtained results confirm the formation of hydrogen bonds between the –O–H and/or –CH groups of KMP and the C=O group of Eudragits. In addition, in the case of KMP-EPO, changes in the dimethylamino groups in EPO suggest that these groups form hydrogen bonds with the C=O/C–O/C–O–C groups of KMP. 

These interactions made it feasible for the formation of an amorphous KMP–Eudragit solid dispersion (confirmed by XRPD analysis). This aligns with other research indicating that the disappearance or shift in vibration peaks is often associated with crystallization or the formation of intermolecular hydrogen bonds [72,73]. Liu et al. [51] proposed a peak shift around 1700 cm^−1^ (C=O), confirming hydrogen bonding between the carbonyl group of EPO and the hydroxyl group of the drug. Similarly, Zong et al. [14] proposed that the ASD of curcumin with Eudragit RS PO and Eudragit RL PO formed a hydrogen bond between the –OH group of curcumin and the C=O group of the polymer. Furthermore, for the curcumin–EPO ASD, the authors suggested that curcumin may donate one hydrogen to the dimethylaminoethyl methacrylate group of Eudragit EPO [14].

The interaction between KMP and Eudragits might affect its solubility. Eudragits are known for their pH-dependent behavior. Specifically, for targeted drug administration in the jejunum, Eudragit L100 is soluble at a pH over 6.0. Eudragit L100-55 is insoluble below pH 5 and soluble above 5.5. Eudragit EPO, on the other hand, is soluble up to pH 5.0 and becomes insoluble above pH 5.0 [74].

The screening involved testing the solubility of the obtained ASDs in four media: water, 0.1 N HCl, pH 5.5 buffer, and pH 6.8 buffer.

It was confirmed that the ASDs of KMP-EL100 exhibited improved solubility in pH 6.8 buffer, and KMP-EPO in pH 5.5 buffer and HCl 0.1 N. Conversely, KMP-EL100-55 showed no improvement in solubility in any of the considered media. Subsequent tests were conducted for selected media. 

Crystalline KMP in pH 6.8, pH 5.5, and HCl 0.1 N buffers was practically insoluble, consistent with reports in the literature [75]. Notably, for KMP-EL100 in pH 6.8 and KMP-EPO in pH 5.5 and HCl 0.1 N, an improvement in solubility was observed compared to pure KMP (Table S6). 

The obtained results show that KMP 40% EL100 exhibited the maximum solubility in pH 6.8, reaching 113.3 ± 2.3 µg·mL^−1^. On the other hand, KMP 50% EPO demonstrated the highest solubility in pH 5.5 (95.1 ± 1.2 µg·mL^−1^), while KMP 30% EPO exhibited the most significant improvement in solubility in HCl 0.1 N (43.5 ± 0.8 µg·mL^−1^).

## 3. Materials and Methods

### 3.1. Materials

Kaempferol (purity > 98%) was purchased from Xi’an Tian Guangyuan Biotech Co., Ltd. (Xi’an, China). Eudragit L100, L100-55, and EPO were purchased from Röhm Pharma (Weiterstadt, Germany). Hydrochloric acid, dimethyl sulfoxide, sodium chloride, and potassium dihydrogen phosphate were obtained from Avantor Performance Materials (Gliwice, Poland). Formic acid 98–100% was purchased from POCH (Gliwice, Poland). A Direct-Q 3 UV purification system (Millipore, Molsheim, France; model Exil SA 67120) was used to produce high-quality clean water.

### 3.2. Preparation of Amorphous Solid Dispersion (ASD) and Physical Mixtures

Amorphous solid dispersions were prepared by the ball milling method at room temperature. Firstly, 500 mg of KMP–Eudragit physical mixtures with different percent content of KMP were obtained by vortexing for 60 s. Next, physical mixtures and three stainless steel balls (diameter 12 mm) were added to a 50 mL stainless steel jar that fit the MIXER MILL MM 400 (Retsch, Haan, Germany). The milling frequency was set to 30 Hz. The milling time was between 30 and 180 min. The total grinding time depended on the success of the KMP amorphization process. The acquired systems had the appearance of being a uniform, fine powder. For further research, the powders were kept in a desiccator.

### 3.3. Determination of the Physical State of Kaempferol in ASDs

#### 3.3.1. X-ray Powder Diffraction (XRPD)

The verification of the kaempferol physical state for (i) the pure compound, (ii) the physical mixture, and (iii) the prepared ASDs was made via powder X-ray diffractometry using a Bruker D2 Phaser diffractometer (Bruker, Germany). The diffraction patterns were captured using CuKα radiation (1.54060 Å) at tube voltages of 30 kV and tube currents of 10 mA. With a step size of 0.02° 2Θ and a counting rate of 2 s·step^−1^, the angular range was 5° to 40° 2Θ. The obtained data were analyzed using Origin 2021b software (OriginLab Corporation, Northampton, MA, USA).

#### 3.3.2. ATR-FTIR Spectroscopy Supported by Density Functional Theory (DFT) Calculations

ATR-FTIR spectra in the MIR region (400–4000 cm^−1^) were obtained using an IRTracer-100 spectrophotometer with a QATR that holds a diamond ATR system (Shimadzu, Kyoto, Japan). The resolution was 4 cm^−1^, while 100 scans over the selected wavenumber range were averaged for each sample. All infrared spectra were collected using LabSolution IR software (version 1.86 SP2, Shimadzu, Kyoto, Japan).

The molecular geometries of KMP were optimized using the density functional theory (DFT) method, employing Becke’s three-parameter hybrid functional (B3LYP) and the standard 6–311G(d,p) basic set. Additionally, calculations for normal mode frequencies and intensities were conducted. The DFT calculations were performed on the PL-Grid platform (website: www.plgrid.pl, accessed on 11 March 2021), utilizing the Gaussian 09 package from Wallingford, CT, USA. The GaussView program (Wallingford, CT, USA, Version E01) was employed to propose the initial geometry of the investigated molecules and visually inspect the normal modes. 

The obtained data were analyzed using the Origin 2021b software (OriginLab Corporation, Northampton, MA, USA).

#### 3.3.3. Principal Component Analysis (PCA)

LabSolution IR software (version 1.86 SP2, Shimadzu, Kyoto, Japan) was used for spectra data preprocessing. Chemometric methods were applied for the purpose of sample discrimination. In the present study, PCA was performed using Origin 2021b software with Principal Component Analysis for Spectroscopy tools (OriginLab Corporation, Northampton, MA, USA). 

### 3.4. Thermal Analysis of KMP—Eudragit Amorphous Solid Dispersions

#### 3.4.1. Thermogravimetric Analysis (TG)

In a TG 209 F3 Tarsus^®^ micro-thermobalance (Netzsch, Selb, Germany), kaempferol, Eudragit L100, Eudragit L100-55, Eudragit EPO, and amorphous solid dispersions were tested for thermal stability. Then, 6–11 mg of the sample powder was added to an 85 µL open Al_2_O_3_ crucible. The TG study’s temperature ranged from 25 °C to 250 °C, with a continuous heating rate of 10 °C per minute in a nitrogen atmosphere (flow rate 250 mL·min^−1^). Proteus 8.0 was used to assess the TG data once collected (Netzsch, Selb, Germany). Origin 2021b software (OriginLab Corporation, Northampton, MA, USA) was used to display the results.

#### 3.4.2. Differential Scanning Calorimetry (DSC)

The differential scanning calorimeter, model DSC 214 Polyma (Netzsch, Selb, Germany), was used to conduct the DSC investigations. Powdered samples weighing 3.9–10.0 mg were placed in sealed pans with lid holes, with the reference sample being a blank sealed aluminum DSC pan with a cover. 

The melting point of KMP in the neat compound was observed using a single heating mode at a temperature range of 30–320 °C with a scanning rate of 10 °C per minute. The glass transition (T_g_) of KMP, EL100, EL100-55 and EPO were observed in melting and cooling modes. A flow rate of 250 mL·min^−1^ was chosen for the nitrogen atmosphere. Proteus 8.0 was used to analyze the acquired DSC data (Netzsch, Selb, Germany). Origin 2021b software (OriginLab Corporation, Northampton, MA, USA) was used to display the results.

Based on the obtained glass transition temperature (T_g_) for KMP, EL100, EL100-55, and EPO, theoretical studies due to the Gordon–Taylor (G-T, Equation (2)) and Couchman–Karasz (C-K, Equation (4)) equations aimed to predict the glass transition value of amorphous KMP–Eudragit systems.

The Gordon–Taylor equation:

(2) $$T_{g,G-T}=\frac{w_{1}T_{g1}+Kw_{2}T_{g2}}{w_{1}+Kw_{2}},$$

where w_1_, w_2_—weight fraction of kaempferol and polymer, respectively. T_g,G-T_, T_g1_, T_g2_—predicted glass transition temperature of a binary system; glass transition temperature of kaempferol, and glass transition temperature of the polymer, respectively. K—constant indicates a measure of interaction between two components. 

K can be expressed mathematically as follows (Equation (3)):

(3) $$K=\frac{\rho_{1}T_{g1}}{\rho_{2}T_{g2}},$$

where $\rho_{1},\rho_{2}$—the densities of two components (KMP: 1.5803 ± 0.0021 g·cm^−3^, L100: 1.2887 ± 0.0004 g·cm^−3^, L100-55: 1.2601 ± 0.0083 g·cm^−3^, EPO: 1.1424 ± 0.0006 g·cm^−3^). The density of KMP, L100, L100-55, and EPO was measured experimentally with a helium gas pycnometer (Accupyc 1340, Micrometrics Instrument Corporation, Norcross, GA, USA). T_g1_, T_g2_—glass transition temperature of kaempferol and polymer, respectively.

Couchman–Karasz equation:

(4) $$T_{g,C-K}=\frac{w_{1}T_{g1}+Kw_{2}T_{g2}}{w_{1}+Kw_{2}},$$

where w_1_, w_2_—weight fraction of kaempferol and polymer, respectively. T_g,C-K_, T_g1_, T_g2_—predicted glass transition temperature of a binary system; the glass transition temperature of kaempferol, and the glass transition temperature of the polymer, respectively. K—constant indicates a measure of interaction between two components.

K can be expressed mathematically as follows (Equation (5)):

(5) $$K=\frac{{∆c}_{p2}}{{∆c}_{p1}}$$

where ∆c_p1_ and ∆c_p2_ is the change in the heat capacity at T_g1_ and T_g2_, respectively.

### 3.5. HPLC Analysis

All HPLC assays were performed on a Shimadzu Nexera (Shimadzu Corp., Kyoto, Japan) [10]. For the stationary phase, a Dr. Maisch ReproSil-Pur Basic-C18 100 column with particle sizes of 5 µm and 100 × 4.60 mm (Dr. Maisch, Ammerbuch-Entringen, Germany) was used. The mobile phase was methanol/0.1% formic acid (70:30 *v*/*v*). A 0.45 µm nylon filter was used to vacuum filter the mobile phase. The experimental conditions were as follows: 0.8 mL·min^−1^ flow rate, 366 nm wavelength, and 35 °C column temperature.

### 3.6. The Solubility Studies of Kaempferol

In this experiment, surplus KMP and ASDs were put into glass vials together with 4 mL of distilled water, pH 6.8 buffer, pH 5.5 buffer, and HCl 0.1 N buffer (pH 1.2). A vortex mixer was used to mix all samples for 30 s (time T0). The samples were then incubated in a lab incubator, the MaxQ 4450 (Thermo Scientific, Waltham, MA, USA), for 24 h at 298.15 K at a steady speed of 75 rotations per minute (rpm). The suspensions were then filtered via a 0.22 µm filter and put through an HPLC analysis procedure. Each measurement was made in triplicate.

## 4. Conclusions

In this study, amorphous dispersions of kaempferol (KMP) with Eudragits were successfully obtained through the milling method. XRPD analysis confirmed that Fourier-transform infrared (FT-IR) spectroscopy combined with principal component analysis (PCA) may serve as an effective analytical tool for verifying the amorphous state of KMP in binary Eudragit dispersions.

Thermal analysis confirmed the complete miscibility of the obtained amorphous dispersions. FT-IR observations indicated the formation of hydrogen bonds between the –O–H and/or –CH groups in KMP and the C=O group in Eudragit.

The amorphization process had a positive impact on the solubility of KMP under different pH conditions. The highest solubility was achieved at pH 6.8 for the dispersion with Eudragit L100 (40% KMP content), at pH 5.5 for the dispersion with Eudragit EPO (50% KMP content), and in 0.1 N HCl for the dispersion with Eudragit EPO (30% KMP content).

## Figures and Tables

**Table 1:** Principal component analysis (PCA); the samples used in the training set and test set.

	Training Set			Test Set		
KMP Content	EL100	EL100-55	EPO	EL100	EL100-55	EPO
20%	A	A/PM	A/PM	ph.m.1		
30%	A/PM	NA/PM	PM	ASD2	ASD5	
40%	PM	NA	A/PM	ASD3	NA4	
50%	NA/PM	PM		ASD		
60%	NA					ph.m.9

Legend: A/ASD—amorphous solid dispersion (confirmed by XRPD); PM/ph.m.—physical mixture; NA/NA4—incomplete amorphous solid dispersion (confirmed by XRPD).

**Table 2:** Summary of the most important parameters of the thermal analysis of the KMP–Eudragit ASD; the experimental and theoretical T_g_ values of KMP–Eudragit ASD.

	Mass (mg)	ΔC_p_ (J·(G·°C)^−1^)	T_g,exp_ (°C)	T_g,G-T_ (°C)	T_g,C-K_ (°C)	Deviation
KMP	6.52	0.519	111.0			
EL100	7.08	0.329	147.1			
EL100-55	5.44	0.336	79.7			
EPO	3.86	0.201	55.4			
KMP 20% EL100	8.28	0.334	128.2	139.4	136.9	N
KMP 30% EL100	5.26	0.391	122.9	135.7	132.5	N
KMP 40% EL100	6.44	0.349	125.7	132.0	128.6	N
KMP 50% EL100	5.28	0.299	126.3	143.6	142.2	N
KMP 20% EL100-55	6.24	0.329	88.9	142.6	137.0	N
KMP 30% EL100-55	6.71	0.334	94.1	140.0	132.7	N
KMP 20% EPO	6.25	0.091	69.1	144.1	132.9	N
KMP 30% EPO	4.63	0.024	78.5	142.3	128.1	N
KMP 40% EPO	3.91	0.122	99.2	140.1	124.3	N
KMP 50% EPO	4.66	0.051	109.3	137.5	121.1	N
KMP 60% EPO	5.18	0.006	117.4	134.4	118.4	N

ΔC_p_—heat capacity; T_g,exp_—glass transition temperature (experimental); T_g,G-T_—glass transition temperature (calculated by Gordon–Taylor equation); T_g,C-K_—glass transition temperature (calculated by Couchman–Karasz equation); N—negative deviation.

## Data Availability

The data are contained within the article and Supplementary Materials.

## Supplementary Materials

The following supporting information can be downloaded at: https://www.mdpi.com/article/10.3390/ijms242417155/s1.
