# Supplementary material for: The Study of Amorphous Kaempferol Dispersions Involving FT-IR Spectroscopy
Source: Int J Mol Sci. 2023 Dec 5;24(24):17155. doi: 10.3390/ijms242417155 (PMC10742969; doi:10.3390/ijms242417155)
Supplement: Supplementary file 1 [file ijms-24-17155-s001.zip › ijms-2680421-supplementary.pdf]

Supplementary Materials

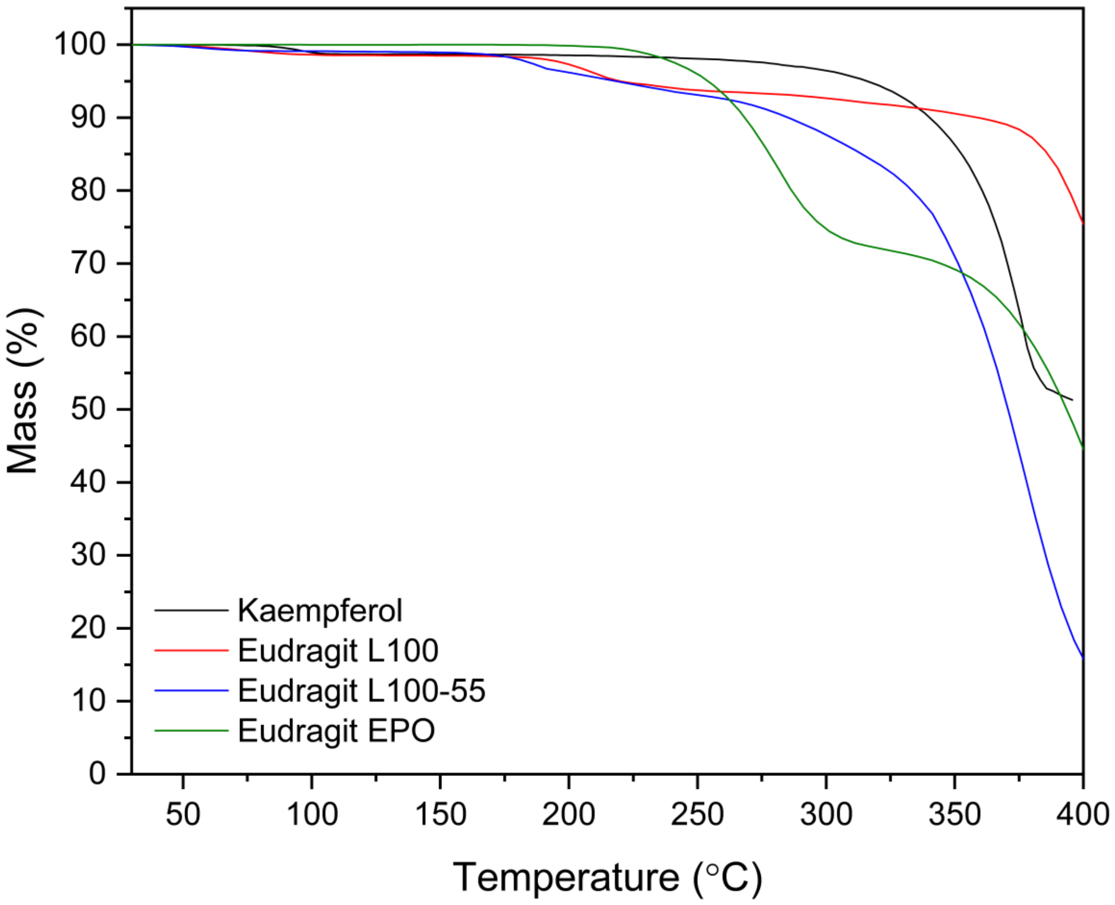

Figure S1. TG analysis: kaempferol (KMP), Eudragit L100 (EL100), Eudragit L100-55 (EL100-55), and Eudragit EPO (EPO).

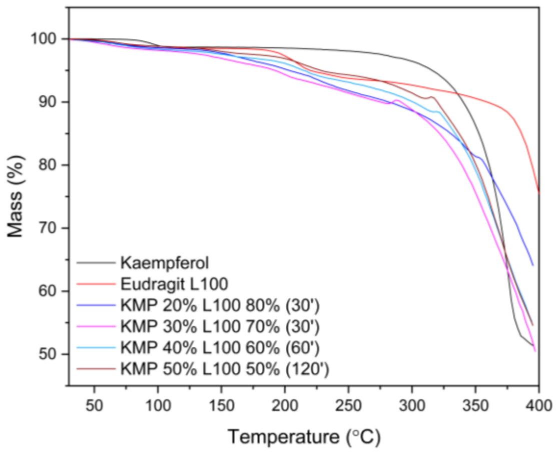

(a)

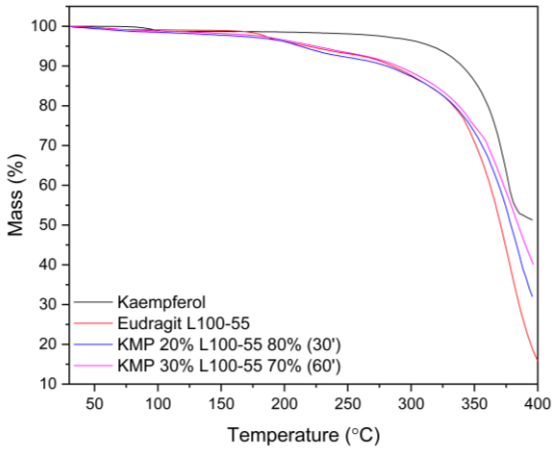

(b)

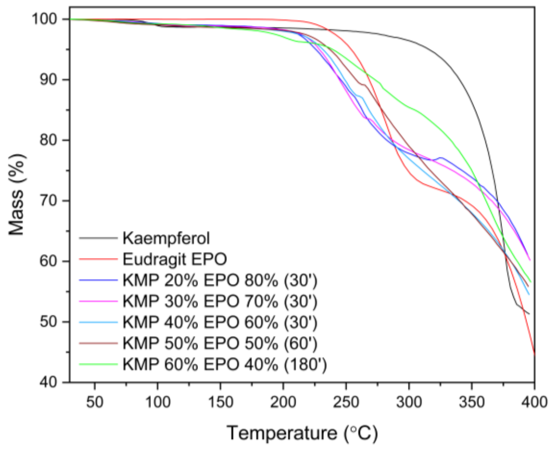

(c)

Figure S2. TG analysis: (a) kaempferol–Eudragit L100 amorphous solid dispersions; (b) kaempferol–Eudragit L100-55 amorphous solid dispersions; kaempferol–Eudragit EPO amorphous solid dispersions.

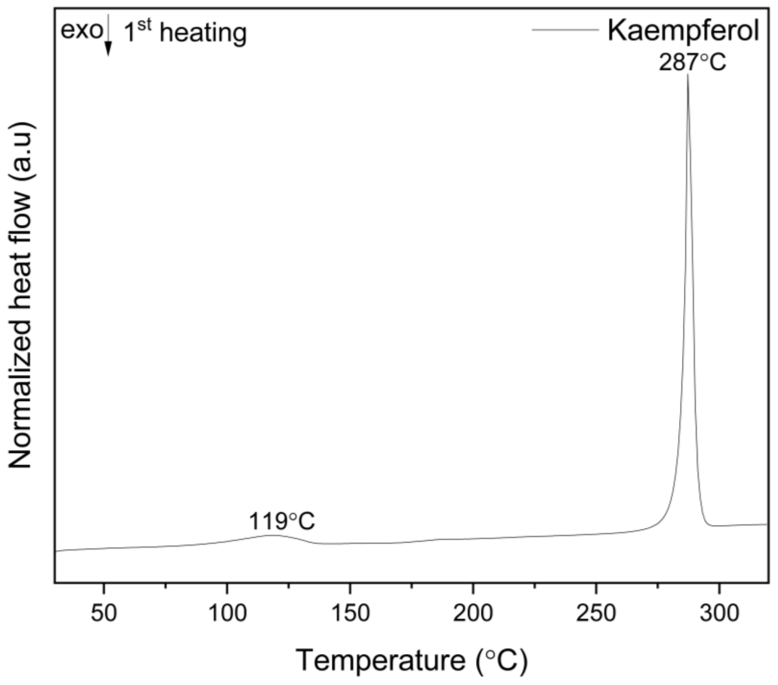

Figure S3. DSC analysis (1st heating): kaempferol (KMP).

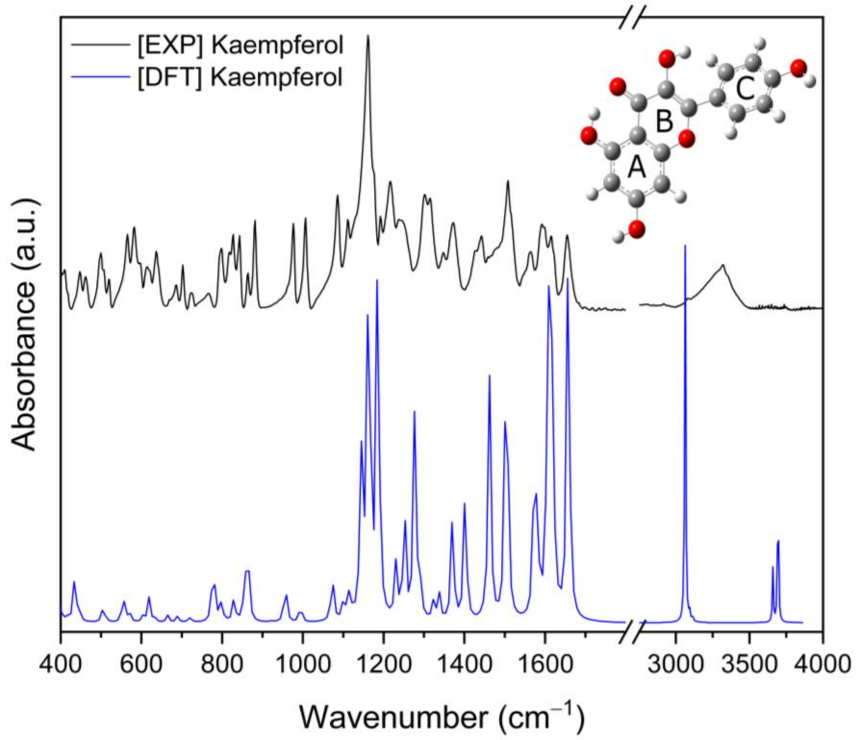

Figure S4. Calculation (DFT) and experimental (EXP) IR absorption spectra of kaempferol at room temperature.

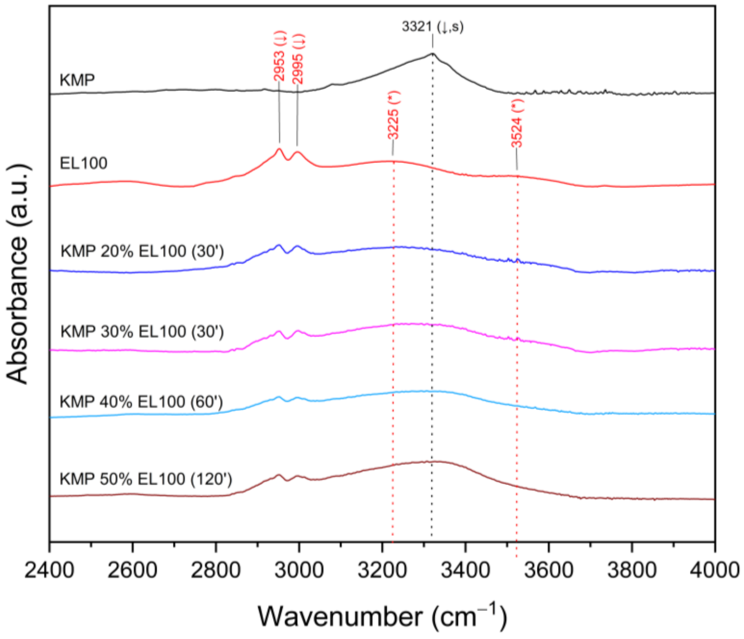
3321 (↓,s)
2953 (↓)
2995 (↓)
3225 (*)
3524 (*)
KMP
EL100
KMP 20% EL100 (30')
KMP 30% EL100 (30')
KMP 40% EL100 (60')
KMP 50% EL100 (120')
Absorbance (a.u.)
Wavenumber (cm−1)
2400
2600
2800
3000
3200
3400
3600
3800
4000

(a)

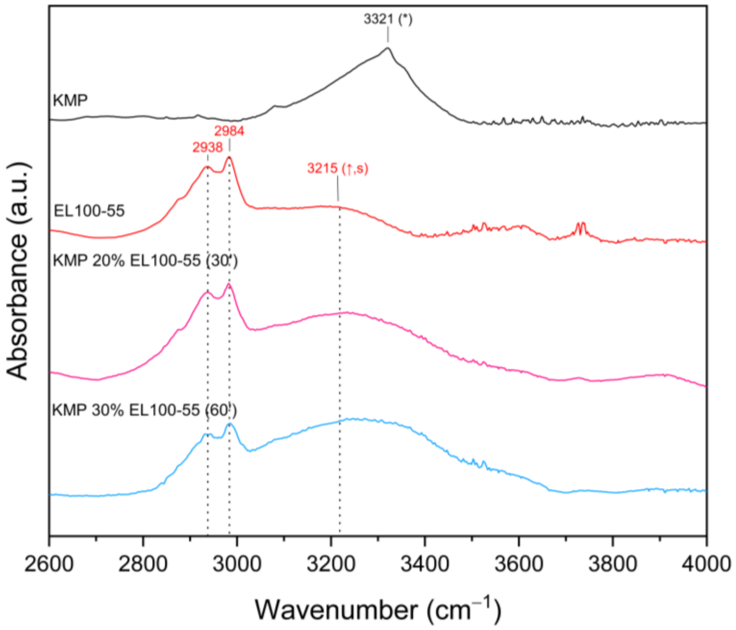
3321 (*)
2984
2938
3215 (↑,s)
KMP
EL100-55
KMP 20% EL100-55 (30')
KMP 30% EL100-55 (60')
Absorbance (a.u.)
Wavenumber (cm−1)
2600
2800
3000
3200
3400
3600
3800
4000

(b)

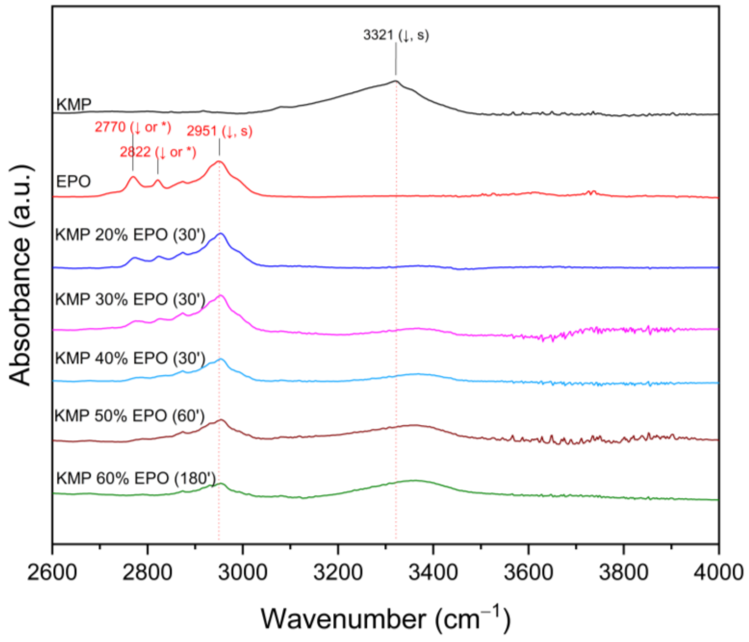

(c)

Figure S5. FT-IR-ATR analysis, range 2600–4000 cm^{-1}: (a) kaempferol–Eudragit L100 amorphous solid dispersions; (b) kaempferol–Eudragit L100-55 amorphous solid dispersions; (c) kaempferol–Eudragit EPO amorphous solid dispersions. Legend: kaempferol (black line, KMP); Eudragit L100/L100-55/EPO (red line, EL100/EL100-55/EPO); the percentage of KMP in the system (KMP 20%–KMP 60%); shape change (#); band disappearance (*); intensity decrease (↓); band shift (s).

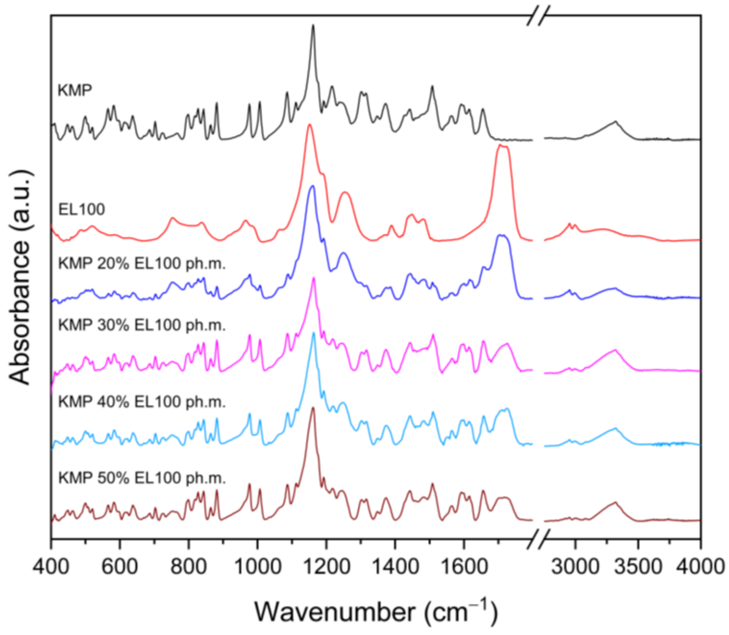

(a)

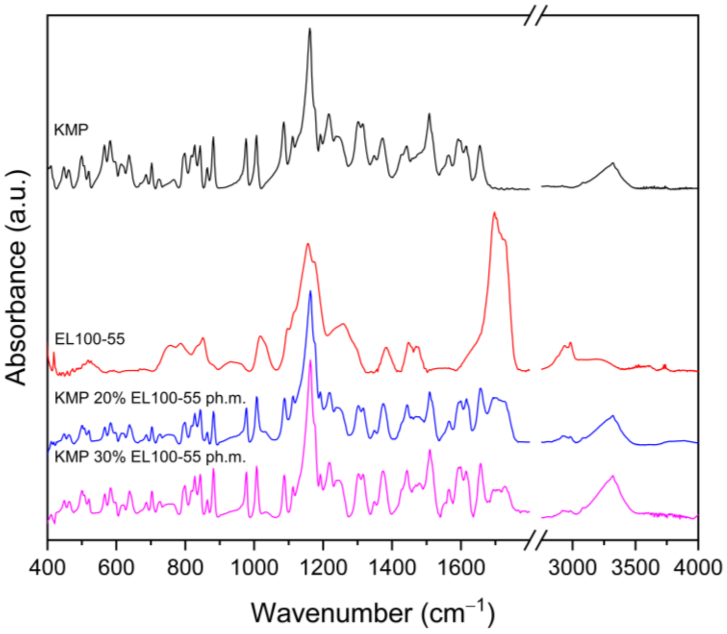

(b)

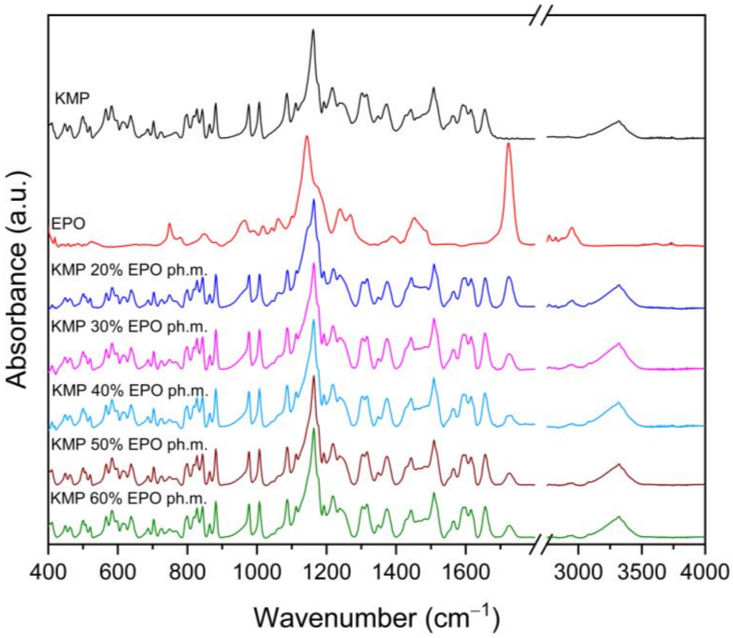

(c)

Figure S6. FT-IR-ATR analysis, range 400–4000 cm^{-1}: (a) kaempferol–Eudragit L100 physical mixture; (b) kaempferol–Eudragit L100-55 physical mixture; (c) kaempferol–Eudragit EPO physical mixture. Legend: kaempferol (black line, KMP), Eudragit L100/L100-55/EPO (red line, EL100/EL100-55/EPO), and the percentage of KMP in the physical mixture (KMP 20%–KMP 60%).

Table S1. Selected characteristic experimental (EXP) bonds (in cm^{-1}) of kaempferol (KMP). Assignments of KMP bands made based on DFT calculations with application of the 6-31G(d,p) basic set.

**Kaempferol (cm^{-1})**	**Assignments**
447	def. all molecule
461	OH w (C)
500	OH w (C)
519	CCOH t (A,B,C) + CCOC t (C) + CCCC t (A,B,C)
565	CCOH t (A,B,C) + CCOC t (C) + CCCC t (A,B,C)
583	CCOH t (A,B) + CCCO t (A,B,C) + CCOC t (C) + CCCC t (B)
617	deformation all molecule
637	CCO b (A) + CCC b (A,B,C) + COC b (C)
702	CCO b (A,B,C) + COC b (C) + CCC b (A,B,C)
723	deformation all molecule
799	CH w (A)
827	CH w (B)
843	CH w (B)
864	OH w (A)
881	OH w (A)
976	CCH b (A) + COH b (A,C)
1007	CCH b (A) + COH b (A,C)
1086	CCH b (A,B) + COH b (A,C) + COC b (C) + CCC b (A,B,C)
1111	CH b (B) + OH b (B)
1217	CH b (A, B) + COH b (A, C) + CCC as s
1302	CO s (A,B,C) + HOC b (A,B,C) + HCC b (B) + CCC b (C,B)
1315	CC s (A,C) + CO s (A,C) + COH b (C,B)
1348	CCC b (A) + COH b (A,C)
1373	CC s (A,C) + CO s (C) + COH b (A,C)
1508	CC s (A,C)
1564	C2=C3s (C) CC s + (A,C) C=O s (C)
1595	C=O s (C) + CC s (A,C)
1616	C=O s (C) + CC s (A,C)
1655	C=O s (C) + C2=C3 s (C) + CC s (A,C)
3321	-OH s

Legend: A, B, C—ring; as—asymmetric; b—bending; def.—deformation; s—stretching; t—torsion; w—wagging.

Table S2. Selected characteristic bonds (in cm^{-1}) of Eudragit L100, Eudragit L100-55, and Eudragit EPO. Assignments bands were made based on values reported in the literature [1,2,11–14,3–10].

Eudragit L100	Eudragit L100-55	Eudragit EPO	
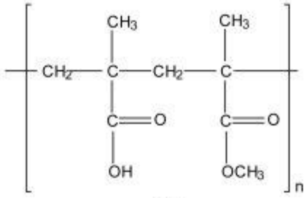 (a)	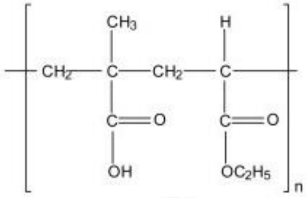 (b)	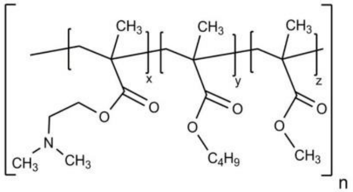	**Assignments**
752			–
839			–
966		964	C–O stretching vibration mode [1] or γ O–H [2]
989		991	C–O stretching vibration mode [1]
1063		1061	C=O stretching vibration [3]
		1144	C–N stretching of aliphatic amine and/or C–O stretching of ester [4] or –C–O–C stretching [5]
1153	1155		–C–O–C stretching [5]/carboxylic ester vibration [6]
1192			C–O vibration of carboxylic acid [7]
		1240	C–O stretching of ester [4,8]
1256	1258		C–O vibration of carboxylic ester vibration [2,6]
		1269	C–O stretching of ester [4,8]
1389			CH_x [9]
1449	1449		CH_3 [10]
		1454	C–H bending of methyl [4]
1481	1474		CH_x [9]
	1697		COO stretching [11]/–C=O stretching vibration [5]
1705			C–O stretching vibration of carboxylic ester [12]
		1722	carboxyl group [13] or C=O vibration [14] of carboxylic ester
1724	1726		C=O stretching vibration groups of carboxylic acids [1,11]
2575	2590		

Table S3. Selected characteristic bonds (in cm^{-1}) of kaempferol (KMP), Eudragit L100 (EL100), and kaempferol–Eudragit L100 amorphous solid dispersions.

KMP (cm^{-1})	EL100	KMP 20% EL100 (cm^{-1})	KMP 30% EL100 (cm^{-1})	KMP 40% EL100 (cm^{-1})	KMP 50% EL100 (cm^{-1})	Assignments
447		*	*	*	*	def. all molecule
461		*	*	↓	↓	OH w (C)
565		↓	↓	↓	↓	CCOH t (A,B,C) + CCOC t (C) + CCCC t (A,B,C)
583		↓	↓	↓	↓	CCOH t (A,B) + CCCO t (A,B,C) + CCOC t (C) + CCCC t (B)
637		↓	↓	↓	↓	CCO b (A) + CCC b (A,B,C) + COC b (C)
702		↓	↓	↓	↓	CCO b (A,B,C) + COC b (C) + CCC b (A,B,C)
799		*	*	*	*	CH w (A)
843		839	839	837	837	CH w (B)
864		*	*	*	*	OH w (A)
	966	*	*	*	*	C–O s vibration mode [1] or γ O–H [2]
976		↓	↓	↓	none	CCH b (A) + COH b (A,C)
1007		↓	↓	↓	↓	CCH b (A) + COH b (A,C)
	1063	*	*	*	*	C=O s vibration [3]
1086		↓	↓	↓	↓	CCH b (A,B) + COH b (A,C) + COC b (C) + CCC b (A,B,C)
1111		*	*	*	*	CH b (B) + OH b (B)
	1153	1159	1161	1159	1159	–C–O–C s [5]
	1192	*	*	*	*	C–O vibration of carboxylic acid [7]
1217		*	1222	1220	1220	CH b (A, B) + COH b (A, C) + CCC as s
	1256	1251, ↓, #	1251, ↓, #	1251, ↓, #	1251, ↓, #	C–O vibration of carboxylic ester vibration [2,6]
1302		*	*	*	*	CO s (A,B,C) + HOC b (A,B,C) + HCC b (B) + CCC b (C,B)
1315		1317, #, ↓	1317, #, ↓	1317, #, ↓	1317, #, ↓	CC s (A,C) + CO s (A,C) + COH b (C,B)
1348		*	*	*	*	CCC b (A) + COH b + (A,C)
1373		1369, ↓	1369, ↓	1368, ↓	1368	CC s (A,C) + CO s (C) + COH b (A,C)
	1389	*	*	*	*	CH_x [9]
	1449	1443	1443	1439	1439	CH_3 [10]
	1481	↓	*	↓	↓	CH_x [9]
1508		1512, ↓	1512, ↓	1512, ↓	1512	CC s (A,C)
KMP [cm^{-1}]	EL100	KMP 20% EL100 [cm^{-1}]	KMP 30% EL100 [cm^{-1}]	KMP 40% EL100 [cm^{-1}]	KMP 50% EL100 [cm^{-1}]	Assignments
1564		*	*	↓	↓	C2=C3s (C) + CC s (A,C) + C=O s (C)

1595		1603, ↓	1603, ↓	1601, ↓	1601, ↓	C=O s (C) + CC s (A,C)
1616		*				C=O s (C) + CC s (A,C)
1655		↓	↓	↓		C=O s (C) + C2=C3 s (C) + CC s (A,C)
	1705	1707, ↓, #	1707, ↓, #	1707, ↓, #	1707, ↓, #	C–O s vibration of carboxylic ester [12]
	1724	1707, ↓, #	1707, ↓, #	1707, ↓, #	1707, ↓, #	C=O s vibration groups of carboxylic acids [1,11]
3321		3269, ↓	3292, ↓	3308, ↓	3331	–OH s

Legend: #—shape change; *—band disappearance; ↓—intensity decrease; A, B, C—ring; as—asymmetric; b—bending; def.—deformation; s—stretching; t—torsion; w—wagging.

Table S4. Selected characteristic bonds (in cm⁻¹) of kaempferol (KMP), Eudragit L100-55 (EL100-55), and kaempferol–Eudragit L100-55 amorphous solid dispersions.

KMP (cm⁻¹)	EL100-55 (cm⁻¹)	KMP 20% EL100-55 (cm⁻¹)	KMP 30% EL100-55 (cm⁻¹)	Assignments
447		*		deformation all molecule
461		*		OH w (C)
500		509, ↓	509, ↓	OH w (A)
519		*		OH w (C)
565		↓		CCOH t (A,B,C) + CCOC t (C) + CCCC t (A,B,C)
583		↓		CCOH t (A,B) + CCCO t (A,B,C) + CCOC t (C) + CCCC t (B)
617		621, ↓	621, ↓	deformation all molecule
702		↓		CCO b (A,B,C) + COC b (C) + CCC b (A,B,C)
723		*		deformation all molecule
799		↓		CH w (A)
827		*		CH w (B)
864		*		OH w (A)
881		↓		OH w (A)
976		↓		CCH b (A) + COH b (A,C)
1007		↓		CCH b (A) + COH b (A,C)
1086		1090, ↓	1090, ↓	CCH b (A,B) + COH b (A,C) + COC b (C) + CCC b (A,B,C)
1111		*	*	CH b (B) + OH b (B)
	1155	1159	1160	–C–O–C s [5]
1194		*	*	OH b (A) + CH b (A)
1217		1221, ↓	1221, ↓	CH b (A, B) + COH b (A, C) + CCC asymmetric s
1244		1252, ↓	1252, ↓	COH b (C) + breathing ring B +COH s (B)
1302		*	*	CO s (A,B,C) + HOC b (A,B,C) + HCC b (B) + CCC b (C,B)
1315		*	*	CC s (A,C) + CO s (A,C) + COH b (C,B)
1348		*	*	CCC b (A) + COH b (A,C)
**KMP (cm⁻¹)**	**EL100-55 (cm⁻¹)**	**KMP 20% EL100-55 (cm⁻¹)**	**KMP 30% EL100-55 (cm⁻¹)**	**Assignments**
	1449	1445	1445	CH_3 [10]
1508		1513, ↓	1513, ↓	CC s (A,C)
1564		*	*	C2=C3 s (C) + CC s (A,C) + C=O s (C)
1595		1601	1602	C=O s (C) + CC s (A,C)

1616		*	*	C=O s (C) + CC s (A,C)
1655		1657, ↓, #	1657, ↓, #	C=O s (C) + C2=C3 s (C) + CC s (A,C)
	1697	1703, ↓	1707, ↓	COO s [11]/–C=O s vibration [5]
	3215	3242, ↑	3267, ↑	O–H [6]
3321		*	*	–OH s

Legend: #—shape change; *—band disappearance; ↓—intensity decrease; A, B, C—ring; as—asymmetric; b—bending; def.—deformation; s—stretching; t—torsion; w—wagging.

Table S5. Selected characteristic bonds (in cm⁻¹) of kaempferol (KMP), Eudragit EPO (EPO), and kaempferol–Eudragit EPO amorphous solid dispersions.

KMP (cm⁻¹)	EPO (cm⁻¹)	KMP 20% EPO (cm⁻¹)	KMP 30% EPO (cm⁻¹)	KMP 40% EPO (cm⁻¹)	KMP 50 % EPO (cm⁻¹)	KMP 60% EPO (cm⁻¹)	Assignments
500		513, #	513, #	513, #	513, #	513, #	OH w (A)
519		*	*	*	*	*	OH w (C)
565		↓	↓	↓	↓	↓	CCOH t (A,B,C) + CCOC t (C) + CCCC t (A,B,C)
583		↓	↓	↓	↓	↓	CCOH t (A,B) + CCCO t (A,B,C) + CCOC t (C) + CCCC t (B)
617		*	*	*	*	*	deformation all molecule
637		#	#	#	#	#	CCO b (A) + CCC b (A,B,C) + COC b (C)
702		↓	↓	↓	↓	↓	CCO b (A,B,C) + COC b (C) + CCC b (A,B,C)
723		*	*	*	*	*	deformation all molecule
799		#	#	#	#	#	CH w (A)
827		#	#	#	#	#	CH w (B)
864		*	*	*	*	*	OH w (A)
881		↓	↓	↓	↓	↓	OH w (A)
976		↓	↓	↓	↓	↓	CCH b (A) + COH b (A,C)
1007		1003, ↓	1003, ↓	1003, ↓	1003, ↓	1003, ↓	CCH b (A) + COH b (A,C)
1086		↓	↓	↓	↓	↓	CCH b (A,B) + COH b (A,C) + COC b (C) + CCC b (A,B,C)
1111		*	*	*	*	*	CH b (B) + OH b (B)
	1144	1146, ↓	1146, ↓	*	*	*	C–N stretching of aliphatic amine and/or C–O stretching of ester [4]
	1240	1242, ↓	*	*	*	*	C–O bond [8]
	1269	1263, ↓	*	*	*	*	C–O bond [8]
1302		1317, #	1317, #	1317, #	1317, #	1317, #	CO s (A,B,C) + HOC b (A,B,C) + HCC b (B) + CCC b (C,B)
1315		1317, #	1317, #	1317, #	1317, #	1317, #	CC s (A,C) + CO s (A,C) + COH b (C,B)
1348		*	*	*	*	*	CCC b (A) + COH b (A,C)
1373		1369	1369	1367	1367	1367	CC s (A,C) + CO s (C) + COH b (A,C)
1508		#	#	#	#	#	CC s (A,C)
1564		1566	1566	1566	1566	1566	C2=C3s (C) + CC s (A,C) + C=O s (C)
1595		1601	1601	1601	1601	1601	C=O s (C) + CC s (A,C)
**KMP (cm⁻¹)**	**EPO (cm⁻¹)**	**KMP 20% EPO (cm⁻¹)**	**KMP 30% EPO (cm⁻¹)**	**KMP 40% EPO (cm⁻¹)**	**KMP 50 % EPO (cm⁻¹)**	**KMP 60% EPO (cm⁻¹)**	**Assignments**
1616		*	*	*	*	*	C=O s (C) + CC s (A,C)

1655		1651	1651	1651	1651	1651	C=O s (C) + C2=C3 s (C) + CC s (A,C)
	1722	↓	↓	↓	↓, #	↓, #	carboxyl group [13] or C=O vibration [14] of carboxylic ester
	2770	↓	↓	*	*	*	C-H s (dimethylamino group) [13]
	2822	↓	↓	*	*	*	C-H s (dimethylamino group) [13]
3321		3366, ↓	3366, ↓	3366, ↓	3368, ↓	3368, ↓	-OH s

Legend: #—shape change; *—band disappearance; ↓—intensity decrease; A, B, C—ring; as—asymmetric; b—bending; def.—deformation; s—stretching; t—torsion; w—wagging.

Table S6. Solubility of kaempferol in amorphous solid dispersion with Eudragit L100 (EL100) and L100-55 (EL100-55).

Medium	Sample	Concentration (μg·mL^{-1})	Improved Solubility (–Fold)
pH 6.8	KMP	-	-
	KMP_20_EL100	10.6±0.8	11
	KMP_30_EL100	29.6±0.6	30
	KMP_40_EL100	113.3±2.3	113
	KMP_50_EL100	76.4±0.9	76
pH 5.5	KMP	-	-
	KMP_20_EPO	34.2±0.2	34
	KMP_30_EPO	40.0±0.5	40
	KMP_40_EPO	64.4±0.9	64
	KMP_50_EPO	95.1±1.2	95
	KMP_60_EPO	62.3±0.3	62
0.1N HCl	KMP	0.7±0.2	-
	KMP_20_EPO	27.5±0.9	39
	KMP_30_EPO	43.5±0.8	62
	KMP_40_EPO	21.0±0.3	30
	KMP_50_EPO	7.3±0.2	10
	KMP_60_EPO	1.6±0.4	2

References

1. Nikam, A.; Sahoo, P.R.; Musale, S.; Pagar, R.R.; Paiva-Santos, A.C.; Giram, P.S. A Systematic Overview of Eudragit® Based Copolymer for Smart Healthcare. *Pharmaceutics* **2023**, *15*, 587, doi:10.3390/pharmaceutics15020587.

2. Dupeyrón, D.; Kawakami; Ferreira; Caceres; Rieumont; Azevedo, R.; Tavares Carvalho, J.C. Design of indomethacin-loaded nanoparticles: effect of polymer matrix and surfactant. *Int. J. Nanomedicine* **2013**, 3467, doi:10.2147/IJN.S47621.

3. Janakidevi, S.; Ramanamurthy, K. V DESIGN OF A NOVEL COLON TARGETED MICROSPONGES LOADED WITH DICLOFENAC SODIUM USING THREE DIFFERENT POLYMERS. *Int. Res. J. Pharm.* **2018**, *9*, 10–19, doi:10.7897/2230-8407.09454.

4. Lin, S.-Y.; Cheng, W.-T.; Wei, Y.-S.; Lin, H.-L. DSC-FTIR microspectroscopy used to investigate the heat-induced intramolecular cyclic anhydride formation between Eudragit E and PVA copolymer. *Polym. J.* **2011**, *43*, 577–580, doi:10.1038/pj.2011.15.

5. Vlachou, M.; Kikionis, S.; Siamidi, A.; Kyriakou, S.; Tsotinis, A.; Ioannou, E.; Roussis, V. Development and Characterization of Eudragit®-Based Electrospun Nanofibrous Mats and Their Formulation into Nanofiber Tablets for the Modified Release of Furosemide. *Pharmaceutics* **2019**, *11*, 480, doi:10.3390/pharmaceutics11090480.

6. Mohammadi, G.; Mirzaeei, S.; Taghe, S.; Mohammadi, P. Preparation and Evaluation of Eudragit® L100 Nanoparticles LoadedImpregnated with KT Tromethamine Loaded PVA -HEC Insertions forOphthalmic Drug Delivery. *Adv. Pharm. Bull.* **2019**, *9*, 593–600, doi:10.15171/apb.2019.068.

7. Santos, T.M.M.; Oliveira Jr, P.H.; Ribeiro, L.A.A.; Oliveira, H.P. Drug/magnetite-loaded enteric particles: the influence of localized magnetic field on controlled release of nifedipine. *Asian J. Biochem. Pharm. Res.* **2014**, *4*, 63–71.

8. Abdelhakim, H.E.; Coupe, A.; Tuleu, C.; Edirisinghe, M.; Craig, D.Q.M. Utilising Co-Axial Electrospinning as a Taste-Masking Technology for Paediatric Drug Delivery. *Pharmaceutics* **2021**, *13*, 1665, doi:10.3390/pharmaceutics13101665.

9. Franco, P.; De Marco, I. Eudragit: A Novel Carrier for Controlled Drug Delivery in Supercritical Antisolvent Coprecipitation. *Polymers (Basel).* **2020**, *12*, 234, doi:10.3390/polym12010234.

10. Kumar, N.; Aggarwal, R.; Chauhan, M.K. Extended levobunolol release from Eudragit nanoparticle-laden contact lenses for glaucoma therapy. *Futur. J. Pharm. Sci.* **2020**, *6*, 109, doi:10.1186/s43094-020-00128-9.

11. Rezazadeh, M.; Safaran, R.; Minaiyan, M.; Mostafavi, A. Preparation and characterization of Eudragit L 100-55/chitosan enteric nanoparticles containing omeprazole using general factorial design: in vitro/in vivo study. *Res. Pharm. Sci.* **2021**, *16*, 358, doi:10.4103/1735-5362.319574.

12. Abdi, M.; Zakeri-Milani, P.; Ghorbani, M. Designing and Evaluating pH-Responsive Electrospun Eudragit® L-100/Hydroxypropyl Methyl Cellulose Composite Mats for Release of Propolis as a Novel Wound Dressing. *J.*

Polym. Environ. **2023**, *31*, 3215–3229, doi:10.1007/s10924-023-02802-4.

13. Sutar, Y.; Nabeela, S.; Singh, S.; Alqarihi, A.; Solis, N.; Ghebremariam, T.; Filler, S.; Ibrahim, A.S.; Date, A.; Uppuluri, P. Niclosamide-loaded nanoparticles disrupt Candida biofilms and protect mice from mucosal candidiasis. *PLOS Biol.* **2022**, *20*, e3001762, doi:10.1371/journal.pbio.3001762.

14. Alshehri, S.M. Versatility of hot-melt extrusion for dosage form design. **2015**.
